# Congenital Toxoplasmosis: Findings in Fetal MRI

**DOI:** 10.7759/cureus.16894

**Published:** 2021-08-04

**Authors:** Claudia Lazarte-Rantes, Raúl Rodríguez-Anccasi, César Rivas-Campos, Elder Silva

**Affiliations:** 1 Pediatric Radiology, Instituto Nacional de Salud del Niño San Borja, Lima, PER; 2 Pediatric Radiology, Resocentro, Lima, PER; 3 Radiology, Hospital Nacional Dos de Mayo, Lima, PER; 4 Radiology, Hospital Nacional Daniel Alcides Carrión, Lima, PER; 5 Radiology, Resocentro, Lima, PER

**Keywords:** congenital toxoplasmosis, fetal mri, abdominal manifestations, toxoplasmosis, cns manifestations, hydrocephalus

## Abstract

*Toxoplasma gondii* infection, when acquired as an acute infection during pregnancy, can have substantial adverse effects on fetuses. We present the case of a 19-year-old pregnant woman with no previous relevant medical history. The ultrasound in the third trimester showed brain and abdominal abnormalities such as congenital hydrocephalus, volume loss of the brain parenchyma, and hepatosplenomegaly. The laboratory test showed positive IgM for *Toxoplasma gondii*. MRI was performed for better assessment and it shows the lesions in the central nervous system and other organs with more details.

## Introduction

Human toxoplasmosis is a cosmopolitan zoonosis caused by *Toxoplasma gondii*. The overall prevalence of acute Toxoplasma infection in pregnant women is estimated at 0.6% (95% CI 0.4e0.7%) and is indicated that annually ~201,600 children are born with congenital toxoplasmosis [1,2]. Humans and other animals develop a systemic infection by the ingestion of contaminated food. Tachyzoites infect nucleated host cells and utilize monocytes, macrophages, and dendritic cells to escape the host immune defense, bypass the blood-brain barrier and the placenta barricade, and spread and form systemic disease [3]. 

Women who have acute toxoplasmosis during pregnancy can transmit organisms to their fetuses through the placenta. Tachyzoites bypass the placental blood barrier and invade the fetal organs to propagate and compromise the embryonic developmental process [3,4].

Several factors affect the risk of mother-to-child transmission, including the gestational age at the time of acute Toxoplasma infection, the virulence of the parasite strain or genotype, the parasite load during acute Toxoplasma infection, and the delay in the initiation of treatment following acute maternal infection [2,5]. The most common congenital manifestation of toxoplasmosis are hydrocephalus, chorioretinitis, intracerebral calcification, mental retardation, and loss of hearing, and very rarely, death may also occur [6,7]. The diagnosis of congenital toxoplasmosis can be made during pregnancy by serological methods with no detectable serum IgG anti-Toxoplasma antibodies but detection of specific IgM and/or IgA and/or IgE antibodies, with low avidity serum anti-Toxoplasma IgG antibodies or seroconversion from IgG negative to IgG positive in cases of sequential testing during gestation [2,8].

We present a case with severe manifestations of toxoplasmosis studied by fetal MRI to assess its compromise in the brain and organs in the abdomen.

## Case presentation

**Figure 1:**
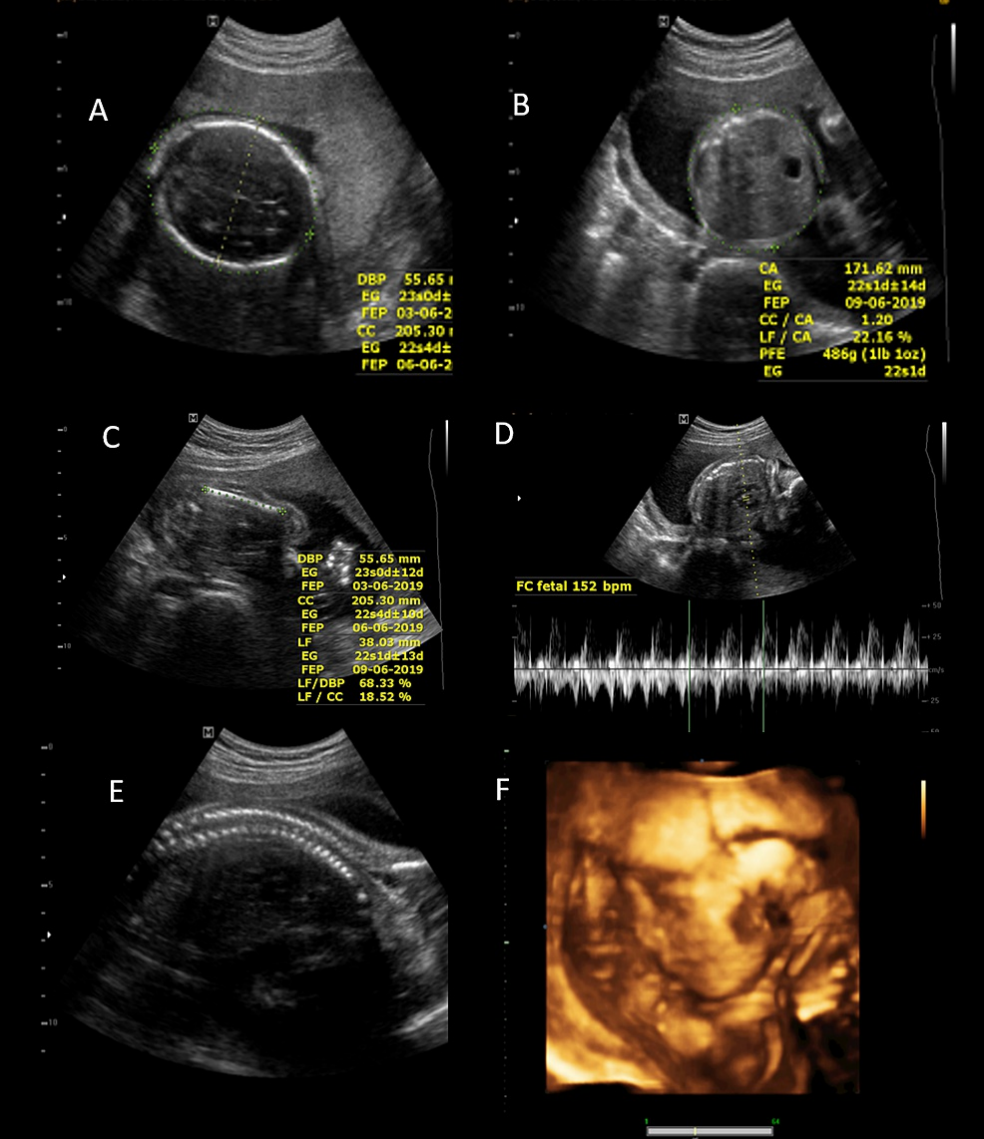
Second-trimester ultrasound Ultrasound in the second trimester showed active gestation of 22ss 3d by biometry with normal fetal morphology. Brain (A), abdomen (B), limbs (C), heart (D), and spine (E) were normal without malformations. 3D ultrasound (F) shows the normal morphology of the face.

The patient is a 19-year-old pregnant woman without any significant clinical history. No alterations were found in the ultrasounds done in the first and second trimesters (Figure 1), and no symptoms were reported.

In the 31st week of gestation, ultrasound showed dilation of lateral ventricles where both lateral ventricles measure 26 mm at the atrium level with no cystic brain changes. No abnormalities were seen in the liver and spleen. The remaining structures demonstrated no morphological alterations.

In the presence of congenital hydrocephalus of an unspecified etiology and with the epidemiological history of contact with cats, she was tested for a TORCH (Toxoplasmosis, Rubella, Cytomegalovirus, and Herpes simplex virus) profile, obtaining positive serology for *Toxoplasma gondii *IgM (> 5000 IU/ml) with low levels of IgG (170.3 IU/ml) and negative serology for Rubeola, Herpes 1-2, and *Cytomegalovirus* (Table 1). Polymerase chain reaction (PCR) in the amniotic fluid was not performed.

**Table 1:** Laboratory testing

Pathogen	Units	Results	Normal values
Toxoplasma Gondii IgG (Elisa method)	IU/ml	170.3	Negative<10 Positive>20
Toxoplasma Gondii IgM (Elisa method)	IU/ml	>5000	Negative<300 Positive>350
Rubeola IgG (Elisa method)	IU/ml	94	Negative<10 Positive>20
Rubeola IgM (Elisa method)	IU/ml	2.9	Negative<2.5 Positive>3.5
Herpes 1 IgG 1 (Elisa method)	IU/ml	6.1	Negative<20 Positive>30
Herpes 2 IgG 2 (Elisa method)	IU/ml	6	Negative<20 Positive>30
Cytomegalovirus IgG (Elisa method)	IU/ml	8	Negative<25 Positive>40
Cytomegalovirus IgM (Elisa method)	IU/ml	1.6	Negative<10 Positive>15

**Figure 2:**
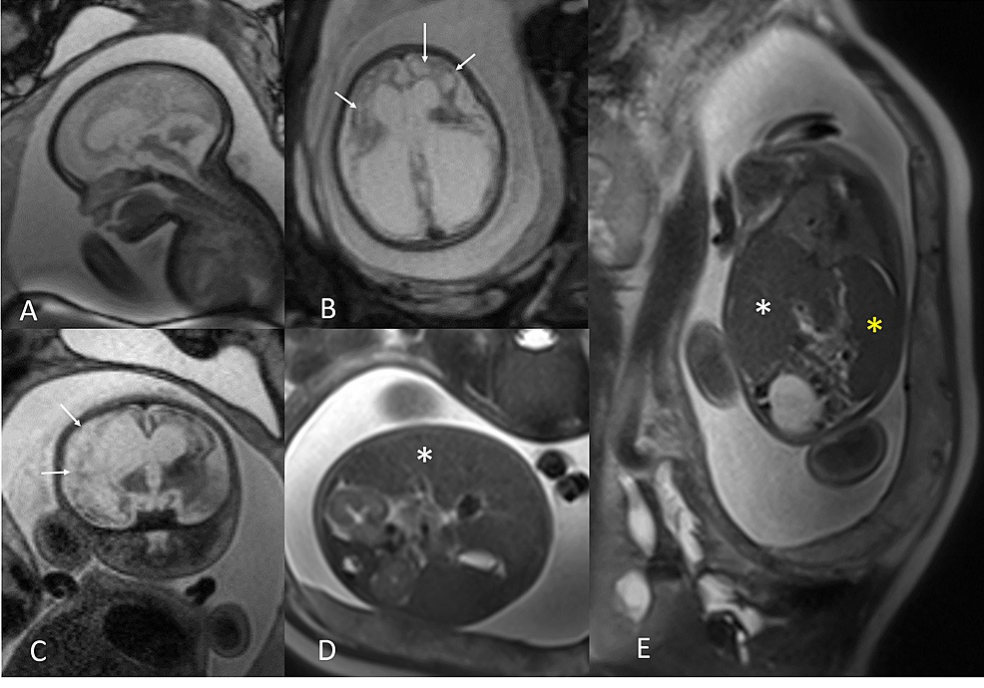
Fetal MRI Sagittal (A), axial (B), and coronal (C) T2-weighted MRI images of the brain show severe loss of parenchymal volume in both cerebral hemispheres with corticosubcortical involvement, both thalamus, basal ganglia, and cerebellum. The cerebral parenchyma is thin with cystic degeneration (arrows in B and C), and there is dilation of the lateral ventricles. Axial (D) and coronal (E) T2-weighted MRI images of the abdomen show the right hepatic lobe reaching the lower pole of the right kidney related to hepatomegaly (white asterisk in D and E) and enlargement of the spleen (yellow asterisk in E).

**Figure 3:**
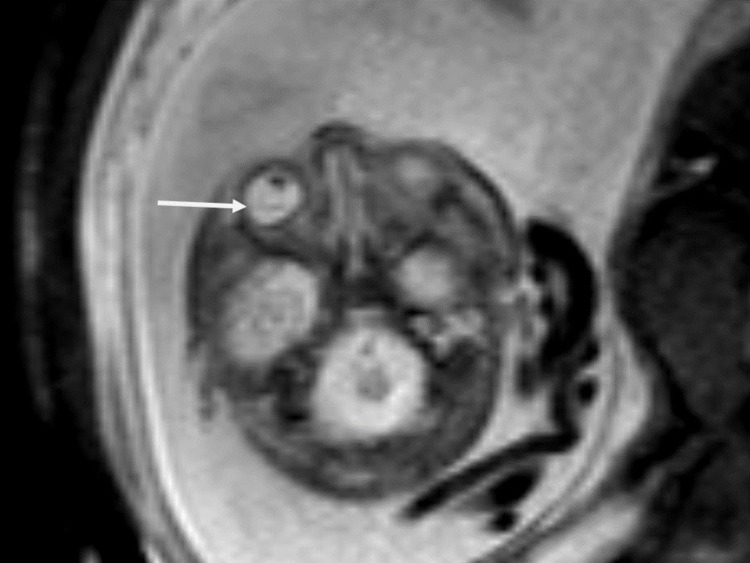
Eye globe abnormalities Axial T2-weighted image at the level of the eye globe shows the detachment of the choroid and retina suggestive of chorioretinitis (arrow).

The MRI performed at 32 weeks of gestation showed the hydrocephalus more evident. Fetal MRI showed a multisystem compromise with severe loss of volume and alteration of the brain and cerebellum that included corticosubcortical cystic degeneration and more dilatation of the lateral ventricles (compared to the previous ultrasound). The right atrium of the lateral ventricle measured 33 mm of transverse diameter and the left 28 mm. The fetal MRI also showed hepatosplenomegaly and signs suggestive of chorioretinitis (Figures 2, 3).

## Discussion

Toxoplasmosis is an endemic disease caused by *Toxoplasma gondii*, a protozoan transmitted to humans by the ingestion of undercooked meat containing the parasite's cysts, contaminated vegetables, or by contact with cat feces [9]. Congenital infection for toxoplasmosis has been found in South America in 3.3 cases per 10,000 [10,11]. The parasite enters the fetus through the placental barrier after primary maternal infection. The risk of fetal transmission increases as gestational age increases but the severity of the disease decreases [12,13].

In our case, the fetal toxoplasmosis infection was diagnosed by serology as part of the prenatal investigation of hydrocephalus, evidenced initially in the ultrasound of the third trimester and the severe damage of the brain and hepatosplenomegaly evidenced in the MRI one week later. The patient had positive IgM with low levels of IgG; other tests for infection were negative (Table 1) [14,15].

The ultrasound findings associated with fetal toxoplasmosis are ventricular dilation, brain calcifications, hepatomegaly, splenomegaly, ascites, pleural effusion, and pericardial effusion [16-18]. In the present case, severe hydrocephalus and polyhydramnios were evident at 31 weeks of gestation, which were not present in previous ultrasound examinations according to the report. A fetal MRI was performed for better assessment of the abnormal findings in the third-trimester ultrasound. The MRI showed loss of the parenchymal volume of both cerebral hemispheres and cerebellum with corticosubcortical cystic degeneration, dilation of the lateral ventricles, and hepatosplenomegaly. Because ultrasound findings can only detect very obvious malformations, other subtle findings can be overlooked; therefore, MRI of the fetal brain is used to better delineate subtle malformations [19]. Differential diagnosis by imaging includes *Cytomegalovirus *[20] and Aicardi-Goutières syndrome; hydrocephaly is also present in aqueductal stenosis, Chiari malformation type 2, and Dandy-Walker malformation, but they are not associated with hepatosplenomegaly. In the present clinical case, MRI is helpful for an accurate evaluation of malformations, providing more significant details of the damage and guiding the diagnosis of fetal pathology with more precision.

In our case, the pregnant woman had performed routine examinations in the first and second trimesters. No pathological findings were found; however, in the third-trimester ultrasound, there was evidence of a significant pathology related to toxoplasmosis, and it was confirmed in greater detail in the fetal MRI. Additionally, the close time difference between the ultrasound and the fetal MRI (one week) showed the rapid evolution of the findings in congenital toxoplasmosis with the development of hepatosplenomegaly and the cystic changes of the brain not seen in the ultrasound.

The limitation of this study is that we could not present the images of the ultrasound performed initially in the third trimester because the patient came from the country's interior and we could access only the report. We could not repeat another serological test for toxoplasmosis three weeks later, as is recommended, due to the demise of the fetus. A PCR of the amniotic fluid was also not performed. However, the laboratory tests done on the mother, epidemiological history, and imaging findings helped make the diagnosis.

## Conclusions

Congenital toxoplasmosis is widespread in developing countries and causes severe damage in fetuses' brains and affects the abdomen, demonstrating its multisystemic compromise. Ultrasound is the initial exam to assess fetal anomalies.

Findings on fetal MRI in affected fetuses establishes a good correlation with the ultrasound findings, helping to determine the impact of the disease on the central nervous system and to assess the involvement of other organs, making fetal MRI an excellent technique to complete the study and derive more information.

Gynecologists, pediatricians, and radiologists need to be aware of imaging manifestations of congenital toxoplasmosis and how MRI imaging can help do a more detailed evaluation of these manifestations.
